# Systemic Mastocytosis Presenting as Pathologic Intertrochanteric Femur Fracture

**DOI:** 10.5435/JAAOSGlobal-D-21-00137

**Published:** 2022-01-11

**Authors:** Aadit Shah, Rohit Bhan, Eduard Praiss Pey, Haley Riordan, Fazel Khan

**Affiliations:** From the Stony Brook University Hospital, Department of Orthopaedics, Stony Brook, NY (Dr. Shah, Riordan and Dr. Khan); and the Stony Brook University Renaissance School of Medicine, Stony Brook, NY (Bhan and Pey).

## Abstract

Systemic mastocytosis (SM) is pathologically characterized by the proliferation of mast cells with infiltrates in various organs, almost always including bone marrow, leading to defects in bone remodeling. Osteoporosis and subsequent fragility fractures are the most common and clinically relevant presentation, although pathologic fracture through the focal lytic lesions can also be observed. Here, we report the case of a 54-year-old woman, with a recent history of unexplained severe allergic reactions, presenting with intertrochanteric fracture of the left femur which on careful history, physical and radiological evaluation was determined to be pathological. The patient was found to have lytic lesions on the CT scan at the fracture site and the pelvis, bilateral femurs, ribs, and sternum, raising suspicion for malignancy. The malignancy workup failed to reveal a primary neoplasm, and the patient was indicated for intramedullary fixation of the left femur along with intraoperative biopsy. Pathologic evaluation of the femoral biopsy was positive for aggregates of mast cells with CD117 (c-KIT, D816V). This finding prompted a bone marrow biopsy, which ultimately led to the diagnosis of aggressive SM. Femoral intramedullary fixation was done with a trochanteric femoral nail, and the patient was postoperatively started on calcium, vitamin D, and physical therapy. Systemic disease was managed by the hematology-oncology team, and the patient was given an epinephrine autoinjector (EpiPen) and managed with midostaurin (Rydapt, Novartis Pharmaceuticals). Treating surgeons should be aware that a pathological long bone fracture can be the initial presentation of SM. Furthermore, surgeons should consider following patients with SM for longer than usual considering a higher risk of complications, such as implant loosening, nonunion, and refracture due to poor and progressively worsening quality of the bone. Our patient was followed with routine visits for 30 months and showed no clinical or radiographical signs of any complications.

Mastocytosis is a rare disorder defined by abnormal clonal mast cell (MC) expansion and accumulation in various tissues, most commonly skin and/or bone marrow. The current World Health Organization classification of mastocytosis includes eight categories under two headings: cutaneous mastocytosis (CM) and systemic mastocytosis (SM).^1^

CM represents 90% of the cases, lacks systemic involvement, is associated with children younger than 2 years, and is commonly characterized by multiple hyperpigmented macular or maculopapular lesions that become urticarial when rubbed or scratched (Darier sign).^2,3^ Subtypes of CM include maculopapular CM, diffuse CM, and mastocytoma of skin.^1^ Diagnosis is made with positive Darier sign, serum tryptase, blood count and differential, and skin biopsy.^3^

SM is associated with adults, infiltrates various internal organs, almost always involves bone marrow, and includes four disease entities: indolent mastocytosis (most common), aggressive systemic mastocytosis (ASM), mastocytosis associated with a hematologic neoplasm, and MC leukemia.^4^ Diagnosis is made with the major criterion of multifocal clusters of abnormal MC in bone marrow and the minor criteria of elevated serum tryptase, abnormal MC CD25 expression, and the presence of the *KIT* D816V mutation.^5^ For patients with suspected SM, comprehensive evaluation of symptoms is recommended, including triggers of MC activation symptoms (ie, heat, friction, NSAIDs, opioids, and anesthetics) and anaphylaxis history after exposure to foods, medications, or other triggers should be particularly noted.^2^

We report a case of ASM presenting as a pathologic left intertrochanteric femur fracture.

## Case Report

A 54-year-old woman presented to emergency department after a fall from standing height resulting in pain of her left hip and an inability to bear weight on the left lower extremity. She reported a medical history of osteopenia intolerant to bisphosphonates but was otherwise in good health. She had a record of routine DEXA (hips and lumbar) 4 months before her presentation that showed osteopenia with a T score of lumbar spine and proximal femur of −1.7. She listed that she was allergic (hives-like reaction) to NSAIDs and was admitted to other hospitals three times just within the past few years for an allergic reaction. Physical examination demonstrated a shortened, externally rotated, and abducted left lower extremity with swelling and ecchymosis of the proximal thigh. No neurologic or vascular deficits were appreciated. The remainder of physical examination and review of systems were within normal limits.

**Figure 1:**
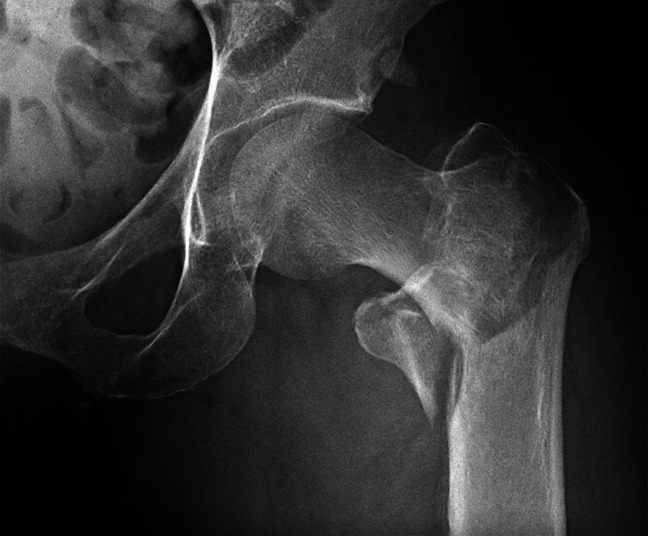
An AP radiograph of the left hip demonstrating an intertrochanteric fracture.

**Figure 2:**
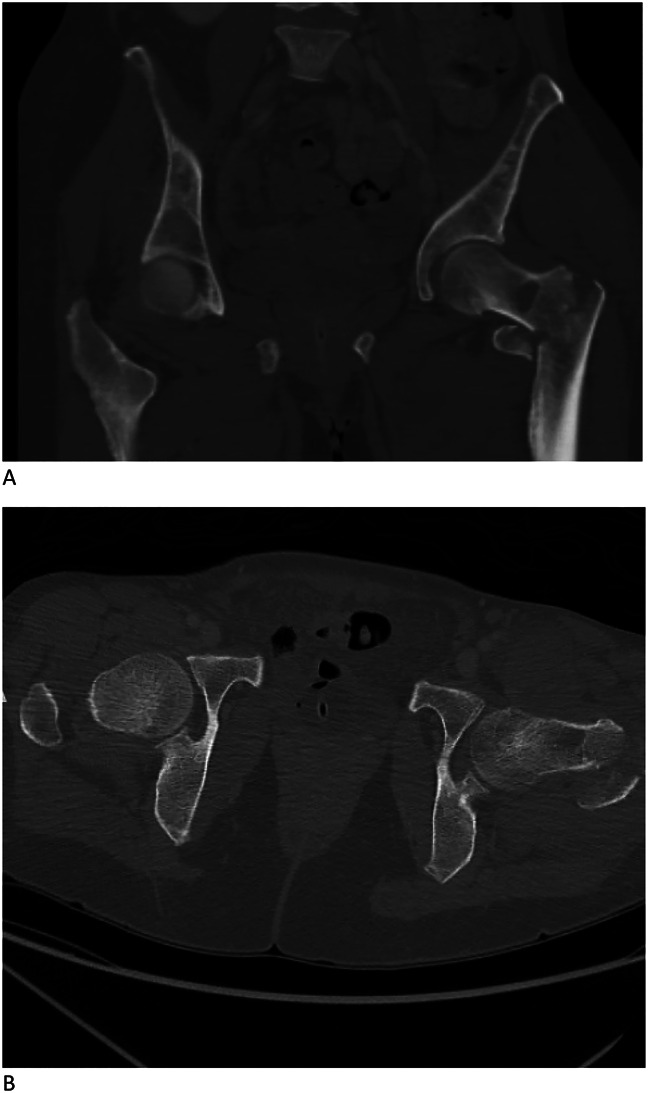
**A** and **B**, Coronal CT imaging revealed multiple lytic lesions in the pelvis (**A**). Axial CT imaging shows an intertrochanteric fracture of the left hip and further demonstrates lytic lesion of the left femur (**B**).

Radiographic evaluation in the emergency department revealed a comminuted and displaced intertrochanteric fracture of the left femur (Figure 1). A CT scan of the hip done for preoperative planning showed unexpected lytic lesions in the ipsilateral femur, and additional lytic lesions in the pelvis and contralateral femur (Figure 2). Because of these suspicious lesions, a full malignancy workup was done, including CT of head, spine, chest, abdomen, and pelvis, as well as SPEP and UPEP, and skeletal survey and bone scan.

In addition to the pelvis and bilateral femur, the CT scan revealed lytic lesions in the ribs and sternum which raised concern for metastatic malignancy and the fracture being a pathologic fracture. She was also found to have chronic compression deformities of L1 and L4 lumbar vertebral bodies. However, no obvious primary visceral malignancy was observed to account for the diffuse skeletal disease. The CRAB criteria helped rule out multiple myeloma because the patient had anemia and bone pain but did not demonstrate hypercalcemia, renal failure, or a protein gap. The SPEP and UPEP results were negative, and a free chain assay demonstrated a mildly elevated kappa:lambda ratio of 1.82 (normal high for our laboratory analysis is 1.65). Her skeletal survey was reported as normal bone density qualitatively without any finding of the lytic lesions. Her bone scan showed radiotracer uptake in the left intertrochanteric region which was consistent with the known fracture along with diffusely mottled appearance of the osseous skeleton that was unspecific in nature. The initial malignancy workup was inconclusive, and the patient was indicated for an open biopsy of the fracture site in addition to intramedullary fixation.

Before surgery, the patient had an episode of hypotension and tachycardia with associated palpitations, as well as flushing and pruritus of the distal extremities bilaterally. This episode resolved with 25 mg IV diphenhydramine and 40 mg IV famotidine and was described by the patient as her typical “allergic reaction” to an unknown trigger.

**Figure 3:**
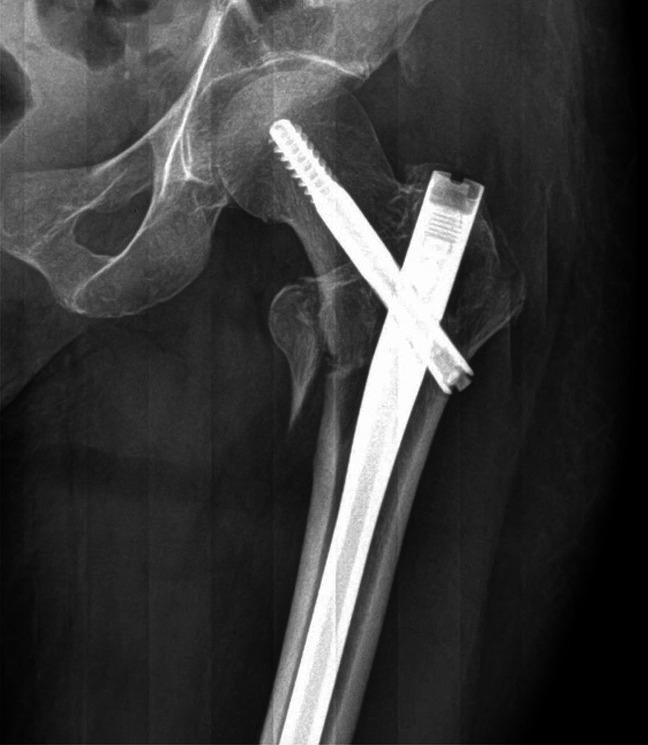
A postoperative AP radiograph of the left hip.

**Figure 4:**
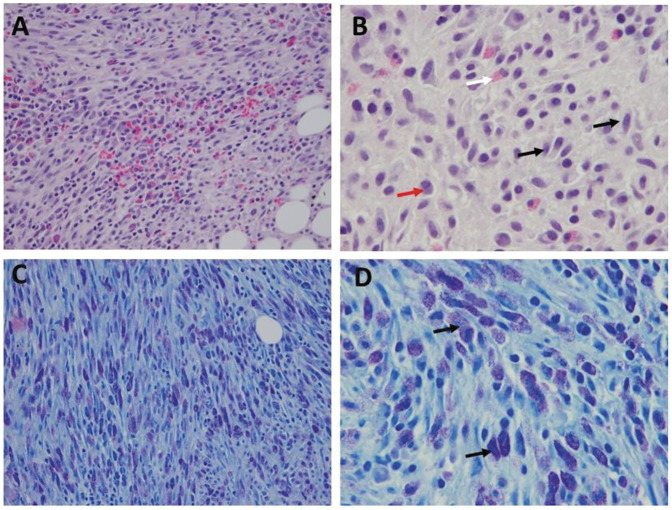
Illustration showing microscopic features and cytochemical characteristics of mast cell disorder: (**A–B**) hematoxylin and eosin-stained bone biopsy from the hip shows large aggregates of spindled (red arrow) and round mast cells (black arrow) and increased eosinophils (white arrow). Most of the mast cells (>25%) are spindle-shaped (**C**–**D**) Giemsa stain showing metachromatic granules (black arrow). In addition, CD117, CD2, and CD 25 immunostains were positive (not shown). The scale bar represents ×400 (**A–C**) and ×100 (**B**–**D**).

The patient underwent the open biopsy and intramedullary fixation with trochanteric femoral nail (Figure 3). Intraoperative frozen sections were inconclusive, requiring a secondary biopsy. Pathologic examination from the biopsy (Figure 4) showed large aggregates of spindled and round MCs positive for CD117 (cKIT) with metachromatic granules observed on the Giemsa stain. Additional immunostaining was positive for CD25 (MCs) and CD2 (T cells). Evaluation for KIT mutation detected a D816V mutation in exon 17. The presence of clonal population of MC in bone marrow, with >25% spindle MC (morphology and immunostains) and detection of cKIT (D816 V) mutation on exon 17 by PCR is consistent with SM. Based on the pathologic findings along with the presence of clinical symptoms (history of pathologic fracture and multiple sites of skeletal involvement), the lesion per pathology team was best classified as ASM (Figure 4). The patient was sent for bone marrow biopsy/aspirate by the hematology oncology team, which, again in conjunction with her clinical picture of allergic reactions to unknown triggers, confirmed the diagnosis of ASM.

**Figure 5:**
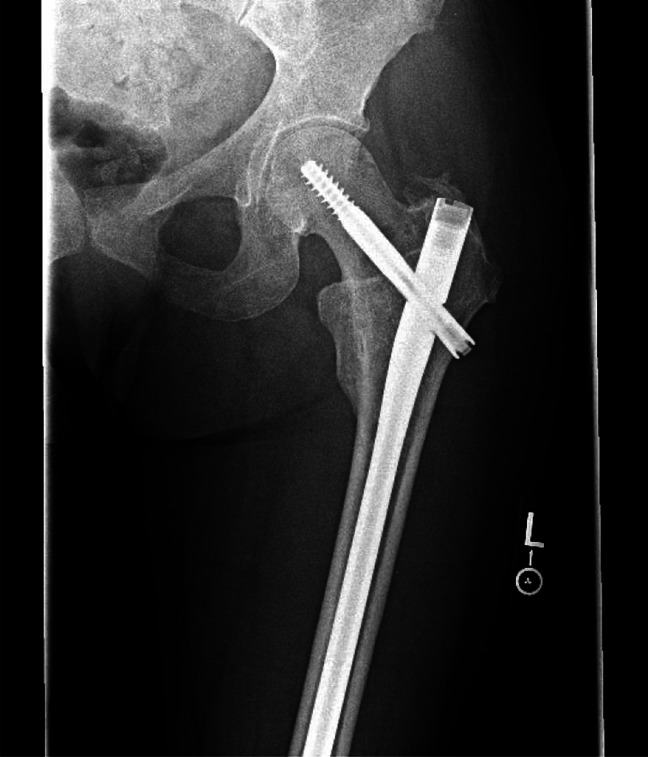
An AP radiograph of the left hip with intact implant at a 30-month postoperative visit.

She was started on calcium and vitamin D and physical therapy after her operation. At 2-week and 1-month visits, the patient was ambulating with a cane and walker, although by the 3-month visit, she was ambulating without any assistive devices. Patient recovered uneventfully and was routinely seen in our orthopedic clinic until she was 30 months postsurgery where radiographs revealed a healed intertrochanteric fracture with intact implant without any complications (Figure 5).

The patient's disease is well managed with midostaurin and an epinephrine autoinjector prescribed by the hematology oncology team, and her bone density is monitored with DEXA. Most recently, a 50-month postsurgery scan showed a T score of −1.8 indicating stable bone density.

## Discussion

Our patient presented with an intertrochanteric femur fracture after a fall from a standing height. With careful history and physical and radiological evaluation, the possibility of this being a pathological fracture was explored, which ultimately led to the diagnosis of SM. One of the most common manifestations of SM in adults is bone involvement. For bone involvement, the spectrum of the clinical picture is wide ranging from fragility fractures due to osteoporosis and pathologic fractures from the neoplastic lesions of the skeleton, bone pain from osteopenia to asymptomatic osteolysis, and foci of diffused osteosclerosis.^6^ Osteoporosis with fragility fractures and pathological fractures remains the most common and the most clinically relevant skeletal presentation. In contrast to the prevailing thought, the more recent epidemiologic studies have shown the prevalence of fragility and pathological fractures and osteoporosis in SM to be as high as 41%.^7,8^ Because of the high prevalence of skeletal manifestations, SM can present through pathological fracture, as was the case for our patient.

Owing to greater impairment of the trabecular bone, SM more commonly presents with vertebral fractures and only rarely with long bone fractures. Fractures in the SM can be attributed to one of the two pathways: pathological fracture at the site of neoplastic lesions or fragility fracture from the osteoporosis and osteopenia. Our case report demonstrates a long bone pathological fracture through a neoplastic osseous lesion which is a rarer form of osseous SM manifestation compared with osteoporosis and vertebral fragility fracture.

Vertebral fractures are markedly more common compared with nonvertebral fractures in SM, and a higher prevalence of osteoporosis and osseous lesions has been demonstrated at the vertebrae than the hip.^9^ One of the reasons for this is that the trabecular bone is affected more than the cortical bone. Direct infiltration of the MCs in the bone marrow causing local bone remodeling disruptions is the most likely reason for greater involvement of the trabecular bone. This neoplastic infiltration of the MCs is one of the two proposed mechanisms that account for bone related changes to SM. First, RANK-RANKL signaling drives the osteoclastic activity and MCs expressing RANK ligand, directly stimulating the osteoclasts leading to increased bone resorption, thereby causing osteolysis and osteoporosis.^10^ This stimulation of the osteoclastic activity by the upregulation of RANK ligand also forms the basis of the osteolytic lesions.^11^ The second mechanism is through the effects of mediators released by MCs. Various cytokines, such as TNF-α, interleukin (IL)-1, 6,17, as well as heparin, histamine, and tryptase, are released by MCs that have direct effects on osteoblasts and osteoclasts.^6,9^ Heparin and TNF-α, IL-1, and IL-6 promote osteoclast activity leading to bone loss causing osteopenia and osteoporosis, whereas histamine has been shown to increase bone formation playing a role in osteosclerosis.^6,9,12^ Osteoporotic involvement in SM is more commonly seen in men compared with women.^7,13^ This suggests that there are hormonal factors that affect how the mediators influence bone remodeling and regulation.

**Figure 6:**
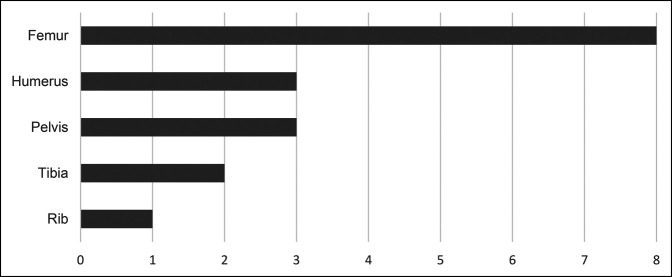
Reported nonvertebral pathologic fracture locations in systemic mastocytosis.

Although less common than vertebral fractures, long bone pathologic fractures in the setting of SM have been reported in the literature. To the best of our knowledge, 17 nonvertebral pathologic fractures in 12 patients have been described in the English literature (Figure 5).^12,14-25^ The chart (Figure 6) reflects the locations of the nonvertebral pathologic fracture described in the literature. In the femur,^12,15-17,19,21-23^ femoral neck (5) was the most common location followed by intertrochanteric (1), proximal shaft (1), and distal femur (1). In the humerus,^24,25^ humeral head (2) and midshaft humerus (1) pathologic fractures have been reported. Acetabulum (2) and inferior pubic ramus (1) comprise the three pathologic fractures reported within the pelvis.^18,21^ Midshaft tibia (1) and medial malleolus (1) are the two reported fractures in the tibia.^14,20^ Of these patients those who needed orthopaedic surgical intervention, the most described complication was nonunion or refracture at the fracture site despite following appropriate surgical and medical treatment measures.^12^ Orthopaedic surgeons must consider the quality of the bone when planning surgical fixation in such cases. In addition, treating surgeons should consider extended postoperative follow-up because of the higher risk of complications, such as implant loosening, nonunion, and refracture, due to the poor and progressively worsening quality of the bone. Our patient was routinely followed with clinic visits postoperatively for 30 months. During the last clinic visit, the patient demonstrated no clinical signs and symptoms of any complications, did not have any hip or femur pain, and was able to ambulate without any restrictions. Radiographs did not demonstrate any signs of nonunion or implant loosening.

Principal medical treatment goal in mastocytosis is the control of mast cells and its mediator-induced symptoms. All patients with SM should be taught to self-administer epinephrine, which could treat life-threatening treating episodes of systemic hypotension or anaphylaxis. H_1_ receptor antagonists, such as loratadine, can be used to control pruritis or flushing. H_2_ antagonists (famotidine) can also be added in the setting of inadequate control . Cromolyn sodium is typically used to provide relief in GI symptoms related to SM.^26^

Osteopenia or osteoporosis related to SM should be treated with appropriate calcium and vitamin D supplementation. Because osteoclasts are primarily responsible for bone loss in SM, disphosphonates have also been recommended.^26^ Unfortunately, patients with SM do not tolerate diphosphonate infusions well and commonly experience worsening digestive symptoms as well as more frequent and severe acute phase responses.^10^ Denosumab, an anti-RANKL monoclonal antibody is currently being studied to treat SM-related bone loss related to SM with positive results.^10^ Palliative radiation therapy has been used for decreasing severe bone pain.^26^

Greater than 80% of the patients with SM have a KIT D816V mutation which drives the disease.^27^ Midostaurin, a multikinase small molecule inhibitor has been shown to have in vitro activity against D816V KIT mutants.^28^ The results of an open-label phase 2 study (CPKC412D2201) using midostaurin in patients with ASM were promising; the overall response rate was 60%, with 45% of the patients demonstrating complete resolution of mastocytosis-related damage in at least one organ system. The median progression free survival was 14.1 months, and the median overall survival was 28.7 months.^29^ Although additional studies are warranted, midostaurin showed efficacy in patients with ASM.

## Conclusion

We present a unique case of SM presenting as pathological intertrochanteric femur fracture. When planning surgical fixation, orthopaedic surgeons should consider bone quality and prolonged follow-up because of an increased risk of postoperative complications, such as nonunion, refracture, and implant failure. Optimal medical management is also imperative in preventing additional bone loss and effective management of the disease (http://links.lww.com/JG9/A184).
